# Solid State Sensors for Hydrogen Peroxide Detection

**DOI:** 10.3390/bios11010009

**Published:** 2020-12-25

**Authors:** Vinay Patel, Peter Kruse, Ponnambalam Ravi Selvaganapathy

**Affiliations:** 1 School of Biomedical Engineering, McMaster University, Hamilton, ON L8S 4K1, Canada; patelv77@mcmaster.ca; 2 Department of Chemistry and Chemical Biology, McMaster University, Hamilton, ON L8S 4M1, Canada; pkruse@mcmaster.ca; 3 Department of Mechanical Engineering, McMaster University, Hamilton, ON L8S 4K1, Canada

**Keywords:** solid state sensors, field effect transistor, chemiresisitive sensor, conductometric sensor, hydrogen peroxide, biosensor and sensors

## Abstract

Hydrogen peroxide (H_2_O_2_) is a key molecule in numerous physiological, industrial, and environmental processes. H_2_O_2_ is monitored using various methods like colorimetry, luminescence, fluorescence, and electrochemical methods. Here, we aim to provide a comprehensive review of solid state sensors to monitor H_2_O_2_. The review covers three categories of sensors: chemiresistive, conductometric, and field effect transistors. A brief description of the sensing mechanisms of these sensors has been provided. All three sensor types are evaluated based on the sensing parameters like sensitivity, limit of detection, measuring range and response time. We highlight those sensors which have advanced the field by using innovative materials or sensor fabrication techniques. Finally, we discuss the limitations of current solid state sensors and the future directions for research and development in this exciting area.

## 1. Introduction

**Figure 1:**
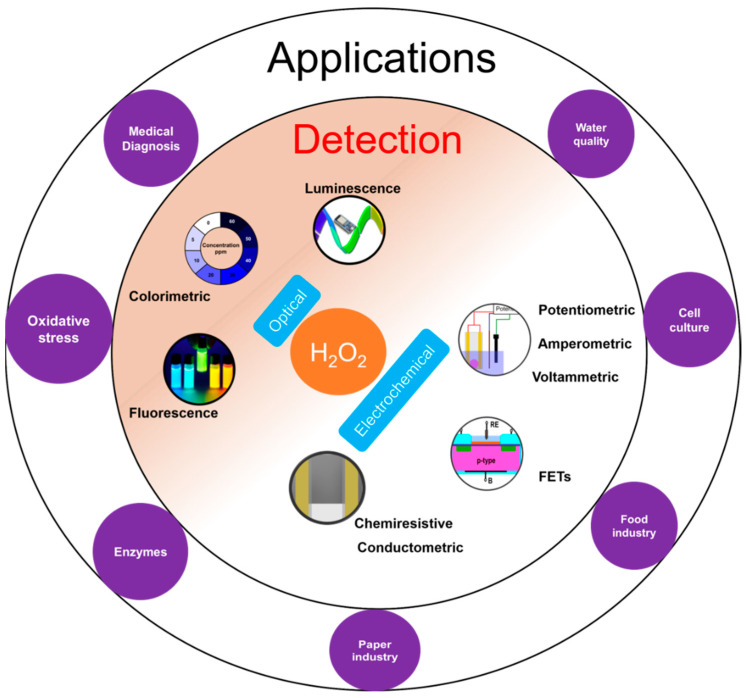
Overview of H_2_O_2_ detection with inner circle containing the two common detection principles: optical and electrochemical; and outer circle with few applications areas of H_2_O_2_ detection.

H_2_O_2_ plays an important role in various applications such as medical diagnostics, clinical research, and industrial sectors like food processing, paper, textile, pharmaceuticals as well as cleaning and disinfection products (Figure 1) [1]. H_2_O_2_ is also important physiologically and is involved in metabolic activities, apoptosis, and immune cell activation [2,3]. It plays an important role as an oxidative stress marker, defense agent, and aging [2,4]. It is a crucial biomarker in monitoring various diseases and disorders including diabetes [5], cancer [6], Parkinson’s [7], cardiovascular, Alzheimer’s [7], and neurodegenerative disorders [7,8]. Moreover, H_2_O_2_ is the intermediate molecule formed in reactions involving numerous oxidases such as glucose oxidase, alcohol oxidase, cholesterol oxidase, lactate oxidase, and glutamate oxidase [9]. Further, H_2_O_2_ is used for sterilizing various medical equipment and residual H_2_O_2_ levels need to be monitored to ensure that the equipment is safe to use [10].

H_2_O_2_ measurement and quantification is performed in a variety of sample matrices including environmental samples like water and soil, human fluids like sweat, blood, cell and tissue cultures. H_2_O_2_ is measured using diverse range of methods such as optical [11,12] including colorimetry, chemiluminescence, and fluorescence; and electrochemical [13,14,15,16] including potentiometry, voltammetry and amperometry (Figure 1). Optical techniques are limited by high cost, complex testing processes, the requirement of sophisticated and bulky instrumentation, need for trained personnel to operate, and interference from sample matrices. On the other hand, electrochemical sensors offer low-cost, simple instrumentation and fast detection [13]. Nevertheless, electrochemical sensors also suffer from a few limitations such as the requirement for a reference electrode, larger working area, etc. The potentiometric method requires a reference electrode for reliable potential measurement while amperometric sensors require a reference electrode to apply a reliable potential bias for the measurement. For potentiometric sensors, a stable response strongly depends on the stability of the reference electrode. However, a miniaturized solid-state reference electrode with long term stability is yet to be realized [17]. For amperometric sensors, a high working electrode potential results in increased interference from interfering molecules [18].

More recently, solid-state sensors such as chemiresistors [19,20,21], conductometric sensors [21,22,23] and field effect transistors (FET) [24,25,26] have been used to measure H_2_O_2_ while avoiding the aforementioned challenges. Chemiresistors consist of a single sensing layer which measures the change in analyte concentration through alteration in resistance of the layer using two contact electrodes. A small potential bias is applied to the substrate film and the change in current is measured. Advantages of chemiresistors are: high sensitivity, because the resistance changes can occur due to modification at any position of the network unlike techniques like colorimetric which is based on volume modifications; ease of fabrication of sensor arrays due to simple sensor structure; suitability for miniaturization; simple instrumentation setup for measurement and elimination of the need for reference electrodes unlike electrochemical methods [27]. FET based solid state sensors are attractive due to their ability to detect analytes with ultrahigh sensitivity. In addition, FETs can be manufactured easily using the established manufacturing process for metal oxide semiconductor FETs (MOSFET) [9].

Previous reviews on sensors for H_2_O_2_ detection have typically focused on electrochemical and colorimetric sensors. Several papers have been published on enzymatic [1,13,14,16,28,29] and non-enzymatic sensing [1,15,30,31] using those principles and the readers are referred to them for an in-depth analysis in these areas. An in-depth review of the emerging class of solid state H_2_O_2_ sensors is not currently available. This review is focused exclusively on chemiresistive, conductometric and FET based H_2_O_2_ sensors which have significant potential for field deployment. A critical analysis of the sensing methods with emphasis on the sensing mechanisms and important parameters like measuring range, limit of detection (LOD), and response time have been provided. The diverse range of functional materials used for sensing and to fabricate these sensors have also been discussed. This review is expected to provide a broad overview of solid state sensors, their suitability for peroxide sensing, and their applications.

## 2. Sensing Mechanism

### 2.1. Chemiresistive Sensors

**Figure 2:**
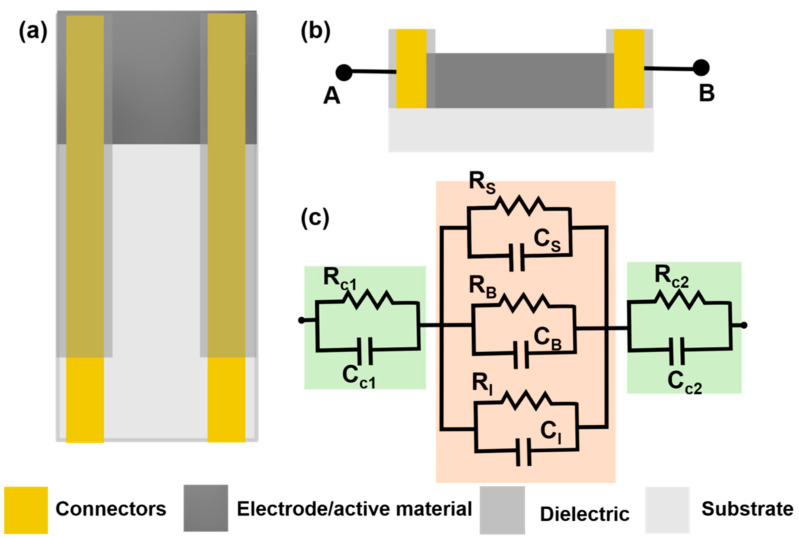
(**a**) Top view of a chemiresistive sensor with 4 main components: active material (dark grey), connectors or contacts (gold), dielectric to insulate the contacts (grey) and substrate (light grey) (**b**) A transverse section of a chemiresistive sensor with two connecting outputs (**c**) An electrical circuit analog for the chemiresistive sensor with R_c1_ and R_c2_, the contact resistance for the first and second contacts, while R_S_, R_B_ and R_I_ are the solution, bulk and interfacial resistance. Similar to resistance, capacitance of all the surfaces are labelled accordingly.

Chemiresistive sensors are a group of sensors which transduces the chemical changes to resistance change. The sensor response is attributed to surface reactions or adsorption of analyte molecules on the sensing film [32]. This type of sensor was originally developed for gas sensing by monitoring resistance changes with adsorption of gas molecules on the sensor surfaces [32,33]. Typically, a sensor is placed under a small potential bias and the change in current is measured as output and converted into a change in resistance. A general chemiresistor consists of four components: the sensitive or active thin film substrate, contact electrodes, passivation layer and substrate (Figure 2a,b). Although for gas sensing, the contacts may be exposed to the environment, they are typically covered with an insulating film to avoid electrical shorting, especially when used in conducting liquids. The equivalent electrical circuit for a chemiresistive sensor can be represented as shown in Figure 2c, where both contacts are represented by parallel RC circuit depicting both Faradaic and non-Faradaic processes. The sensing layer which remains in contact with the solution is divided into three parallel RC circuits representing surface, bulk, and interface processes. When chemiresistive sensors are operated in DC mode, all capacitance can be neglected from the equivalent circuit (Figure 2c).

During measurement, the sensor is exposed to the analyte, and adsorption of analyte to the active thin film results in a change in resistance. For instance, carbon nanotubes (CNTs) are generally p-doped when the films are coated using water based CNT dispersions and if analyte adsorption results in the release of electrons, the hole concentration in the active surface is reduced, which results in a decrease in resistance [34]. On the other hand, if the analyte extracts electrons from the CNT film, this will lead to an increase in dominant carriers resulting in an increase in conductivity. Further, this change in resistance due to analyte interactions can occur from factors like increasing the CNT-CNT junction resistance modulation of the Schottky barrier at the CNT-metal contact junction and charge transfer between analyte and CNT. These processes have been described in detail in other reviews [35,36]. These sensors have some limitations such as irreversible changes introduced onto the substrate due to application of a potential bias, a high dependence of the sensitivity of the sensor on the substrate thickness, and high contact resistance which can further reduce the sensitivity of the sensor. For instance, in the case when conducting polymers are used as the functional sensing layer, the potential bias can induce an irreversible change in the polymer film resulting in a change to the baseline resistance of the sensor. The analyte can also cause irreversible changes to the sensors surface [19]. Thinner films generally have higher sensitivity as compared to thicker films [33]. For two point measurements, the resistance change has two components: change due to the analyte binding and change in contact resistance between the substrate and the metal contacts [37].

### 2.2. Conductometric Sensors

Conductometric sensors are devices which detect the change in conductivity of the analyte solution due to consumption or generation of ions due to chemical reactions using two conducting electrodes [38]. This method was originally developed to study chemical kinetics of reactions and later exploited by researchers to detect enzyme catalyzed reactions. Conductometric measurements are non-specific as conductivity changes can occur due to the migration of all ions present in the solution. This non-specificity is circumvented by coating enzyme on top of the electrode and doing the measurements in a defined measuring cell. Conventionally, the conductivity measurements are performed in AC mode. Unlike chemiresistive sensors, these sensors offer information through the frequency of the measurement, an important experimental variable to determine non-Faradaic processes. An alternating bias has several advantages such as minimized contact polarization, double layer charging and electrode polarization [39].

**Figure 3:**
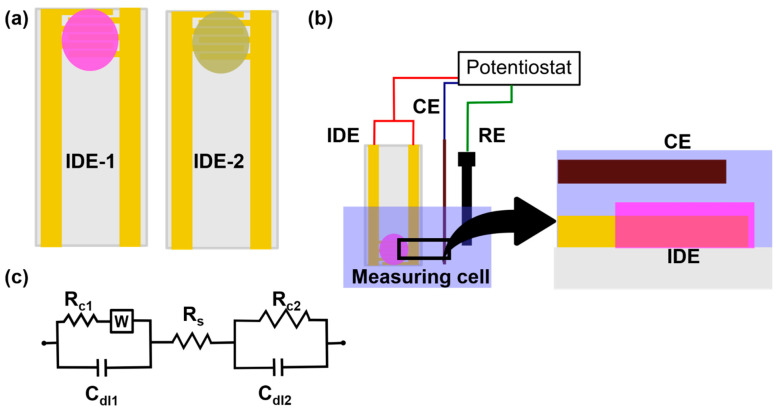
(**a**) A pair of interdigitated electrodes (IDE): IDE-1 represents the active membrane coated electrode and IDE-2 is the electrode coated with membrane without any active material (**b**) A schematic representing the experimental setup of a conductometric sensor with IDE, a counter electrode and a reference electrode. The zoomed in picture shows a transverse section of IDE and counter electrode (**c**) An equivalent circuit for the conductometric sensor with R_C1_, W and C_dl1_ representing the charge transfer resistance, Warburg impedance and double layer capacitance, respectively for the IDE; R_S_ is the solution resistance; and R_C2_ and C_dl2_ representing the charge transfer resistance and double layer capacitance for the counter electrode respectively.

Typically, conductometric measurements are done using a pair of identical electrodes (generally interdigitated electrodes) dipped in a solution container with a constant volume. One of the interdigitated electrodes (IDE-1) is coated with the enzyme film and the other does not have any enzyme layer (IDE-2) (Figure 3a). The IDE-2 determines the base conductivity response from other ions and molecules present in the solution. The measurement of both the sensors are done with respect to a counter and/or reference electrode (Figure 3b). The final sensor response is determined by subtracting the signal of IDE-1 from the signal of IDE-2. Here, the impedance is measured perpendicular to the electrode surface. The equivalent circuit of the electrochemical cell is shown in Figure 3c where R_ct1_ and R_ct2_ are the charge transfer resistances for IDE and CE respectively, W is the Warburg impedance for the IDE which models the diffusional resistance due to both interfaces, C_dl1_ and C_dl2_ are double layer capacitances of IDE and CE respectively and R_s_ is the solution resistance. Enzymatic conductometric sensors are versatile sensors which are low-cost, need a smaller potential bias and require simple instrumentation to generate reliable signals. However, the sensing signal can be affected by temperature variations [39] and changes in the ionic strength of the solution.

### 2.3. FET

**Figure 4:**
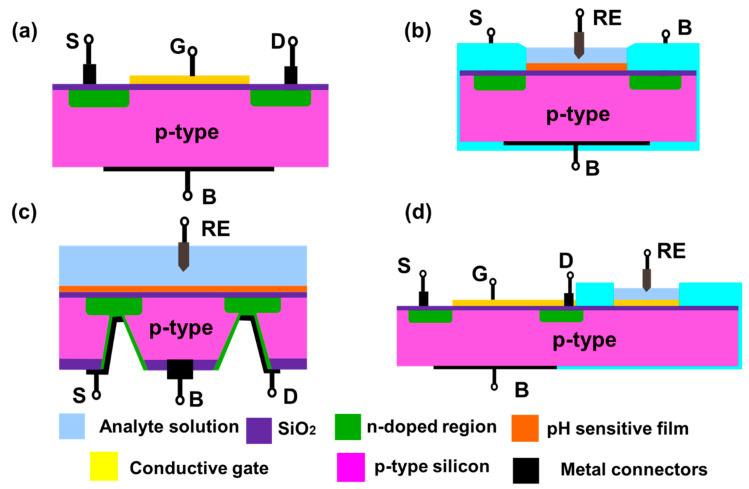
Schematics of a (**a**) MOSFET with p-type silicon as a base substrate (pink) and n-doped source and drain region (green) (**b**) ISFET with a pH sensitive film (orange) (**c**) Back gated FET with analyte solution and RE on the top side and all the terminal connections are done from the back side (**d**) Extended gate FET with a regular MOSFET and an extended sensing region connected with gate terminal of the MOSFET. S, G, D and B are source, drain, gate and base substrate terminals (all shown in black). RE is the reference electrode.

Metal oxide field effect transistors (MOSFETs) are used in electronic circuits as switches, gates, amplifiers etc. MOSFETs can be three or four terminals depending on the presence or absence of back gate (base substrate): source, drain, gate and base substrate. Insulated gate FET (IGFET) is the most common type of MOSFET used currently for chemical sensing. The gate terminal of the IGFET is insulated using a dielectric layer (like SiO_2_). A typical n-channel FET is constructed using a p-type substrate with heavily doped n-type source and drain (Figure 4a). The operation of the FET depends on the potential bias applied to the gate. Under zero bias, the FET channel is non-conducting. For n-channel FET, the conduction begins after a critical threshold potential is applied to the gate. This threshold potential will induce an inversion layer.

Early H_2_O_2_ (and glucose) FET sensors had pH sensitive material coated on the gate insulator that made it sensitive to changes in local pH due to generation or consumption of hydrogen ions by an enzyme that catalyzes H_2_O_2_ (Reaction 1) [40,41,42]. In this reaction, the reduction of H_2_O_2_ was catalyzed in presence of horseradish peroxidase (HRP), with the iodide ion acting as a reducing agent [43].

(1) $$H_{2}O_{2}+2I^{-}+2H^{+}\overset{\mathrm{HRP}}{\to }{\;I}_{2}+2H_{2}O\;$$

In such FETs, the gate dielectric is converted into a hydrogen sensitive film which can generate similar potential change in presence of the analyte (Figure 4b). Then the channel conduction can be influenced by changes in the hydrogen ion concentration. These devices are known as ion selective FETs (ISFETs). Similar to MOSFETs, ISFETs can also be n-channel or p-channel ISFET depending on the doping of the silicon substrate used to fabricate the FET. The drain current depends on the resistance of inversion layer and, the potential applied between source and drain. Mathematically, the drain current (*I_d_*) of ISFET is given by [9]:

(2) $$I_{d}=\mu C_{i}\left(\frac{W}{L}\right)V_{d}\left[V_{g}-\left(E_{ref}-\phi +\chi_{sol}-\left(\frac{\phi_{Si}}{q}\right)-\frac{Q_{i}+Q_{ss}}{C_{i}}-\left(\frac{Q_{b}}{C_{i}}\right)+2\phi_{f}\right)-0.5V_{ds}\right]$$

where *μ* is mobility of electrons in the channel; *L* and *W* are length and width of the channel, respectively; *q* is the elementary charge, *Q_b_*, *Q_i_*, *Q_ss_* are the charges located in depletion region, insulator region, and surface and interface states, respectively, *χ_sol_* is solution’s surface dipole potential, *E_ref_* is the reference electrode’s potential, *ϕ_Si_* is the electron work function of silicon, *ϕ_f_* is the potential difference between Fermi level of doped and intrinsic silicon, *V_ds_* is the potential applied to the drain with respect to source, *V_d_* is the drain potential, *V_g_* is gate potential, *C_i_* is the capacitance value of the gate, and *ϕ* is the potential of membrane–electrolyte interface.

Commonly used pH sensitive materials for ISFETs are silicon nitride (Si_3_N_4_), aluminum oxide (Al_2_O_3_), and tantalum oxide (Ta_2_O_5_) [9,40,41,42,44,45,46]. pH sensitivity is a surface phenomenon where the surface hydroxyl groups interact with the protons. The consequent changes in the surface charge or potential of the gate material leads to a current flow in the channel. This generated current is proportional to the analyte concentration. ISFETs are increasingly popular due to advantages such as rapid detection, small size, established manufacturing process, easy integrability in arrays, and their ability to be stored in dry form. However, they suffer from higher drift as compared to ion selective electrodes.

Based on its design, the ISFETs can be front side and back side connected. Front side ISFETs are widely used due to ease of fabrication, but they make it difficult to passivate the device from the analyte solution. Back side ISFETs solve the passivation problem as all the connections are accessed from the back side of the silicon chip. However, it poses a manufacturing difficulty to etch a deeper cavity into the silicon chip for connecting the source and drain from the back side of the chip (Figure 4c). An alternate configuration known as an extended gate FET was proposed in 1983 [47] (Figure 4d). The device has two components: a MOSFET with electrical connections and an extended gate with a pH sensitive film. This device has advantages such as low manufacturing cost due to simpler fabrication and packaging, and long term environmental stability of the device as the FET is not directly exposed to the solution.

## 3. Chemiresistive Sensors

Chemiresistive sensors were initially developed to detect gases or vapors but in the past two decades several chemiresistive sensors have also been developed to measure analytes in liquid environments. These sensors consist of two main components: an active thin film and electrical contacts (Section 2.1). One of the first H_2_O_2_ chemiresistive sensor was fabricated from polypyrrole and multiwalled CNT (MWCNT) [48]. Chemiresistive sensors can be broadly classified based on active sensing thin film material. The active thin film can be made of various conducting or semiconducting materials such as CNTs [19,48,49], conducting polymers [20,50,51] or combinations of these materials. Contact electrodes are made of conductive materials including conductive carbon [50,51], metal electrodes like platinum [48], gold [19,20,52], and silver [49]. The sensors are compared based on three parameters: measuring range, LOD and response time. A summary of H_2_O_2_ chemiresistive sensors is given in Table 1.

### 3.1. Chemiresistive Sensors Based on CNTs

CNTs are widely used for fabricating sensing surfaces due to their superior transduction as well as electrical and mechanical properties. Some of the features offered by CNTs include good sensitivity to change in resistance due to analyte binding, high surface-area to volume ratio and good electrical conductivity. However, CNTs pose some challenges like poor solubility in common solvents resulting in unstable dispersions which may affect the reliable fabrication of these sensors. Therefore, several dispersing molecules like sodium dodecylbenzenesulphonate (NaDBS) [48] or poly(4-vinylpyridine) (PVP) [19] have been investigated to stabilize the dispersions and facilitate reliable fabrication such as in the case of glucose detection when coated with glucose oxidase (GOD).

**Figure 5:**
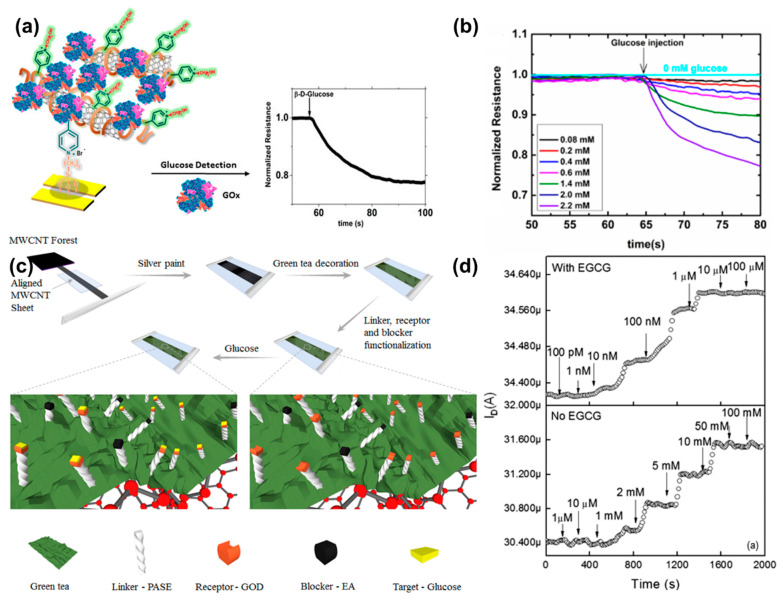
(**a**) A chemiresistive glucose sensor with GOD immobilized on a MWCNT-PVP film. A reduction in resistance is observed when the sensor is exposed to the glucose concentrations. Reprinted with permission from [19]. Copyright 2017 American Chemical Society. (**b**) Normalized resistance change of the sensor shown in Figure 5a, when it is exposed to glucose concentrations ranging from 0.08 mM to 2.2 mM. Reprinted with permission from [19]. Copyright 2017 American Chemical Society. (**c**) Schematics showing the steps for sensor fabrication with green tea and MWCNT and GOD. Reprinted from [49] with permission from Elsevier. (**d**) The current response for the sensor depicted in Figure 5c with and without EGCG. Reprinted from [49] with permission from Elsevier.

One of the first chemiresistive sensors prepared using CNTs used NaDBS as the dispersant to stabilize the colloid. The sensing layer was a composite of CNTs, polypyrrole and NaDBS. Here, NaDBS acted as a dopant for polypyrrole and dispersant for MWCNTs (described in Section 3.2) [48]. The sensor exhibited a measuring range from 0–20 mM for H_2_O_2_. The study demonstrated a chemiresistive sensor fabricated using CNTs and polypyrrole, but it did not investigate the effect of interferent species on its response. The sensor was also found to be sensitive to environmental parameters such as temperature and pH. Another CNT based chemiresistive sensor was fabricated using a composite containing SWCNTs and PVP [19] (Figure 5a). PVP was used as the dispersing agent and the polymer also offers nucleophilic sites (pyridyl nitrogen) for electrostatic modification of the layer. GOD was drop casted followed by treatment with glutaraldehyde to crosslink the enzyme. According to previous literature reports, glucose in presence of oxygen is catalyzed by GOD to generate gluconic acid and H_2_O_2_ [48]. Glucose was detected by measuring the increase in current due to the generation of H_2_O_2_. The detection was performed under a potential bias of 0.1 V at pH 5.5 with a linear range from 0.08–2.2 mM and 0.08 M as the LOD (Figure 5b). The sensor did not respond to interferents such as fructose and sucrose, but other potential interferents such as ascorbic acid, uric acid, were not included in the study. The sensor had a quick response time of 3 s. However, a large variation of ~18.9% was found between different sensors indicating a need to standardize the fabrication process. The study also reported that peroxide caused irreversible change on the sensor which limited its use to 5 times. The sensor retained 83.3% of the initial response after 45 days of storage at 4 °C.

Apart from CNT dispersions, CNTs can be transferred from the grown surface to the substrate. Here, the sensor was fabricated on a PET sheet and a chemical vapor deposited CNT film was transferred to it [49]. A reactive oxidative species sensitive molecule, epigallocatechin gallate (EGCG), a compound found in green tea, was coated on the MWCNT film. GOD was immobilized using pyrene butanoic succinimidyl ester where the pyrene ring interacts with EGCG, and the succinimidyl ester forms an amide bond with GOD (Figure 5c). The sensor measured glucose concentrations from 10 nM to 10 μM with a LOD of ~8.7 nM when operated under a constant potential bias of 100 mV. In absence of EGCG, the dynamic detection range increases to 1–10 mM (Figure 5d). Without EGCG, the H_2_O_2_ molecules directly p-dope the CNT film and change the resistance. However, in presence of EGCG, direct doping by the H_2_O_2_ molecules is limited. The change in resistance is mainly due to oxidation of EGCG by H_2_O_2_ and a subsequent p-doping shifts the detection range to a lower concentration range. No significant interference was observed in presence of acetaminophen, ascorbic acid, and uric acid. Even though the sensor showed an estimated response time of ~400 s which was higher as compared to the PVP-CNT sensor [19], it can be used to detect H_2_O_2_ in sub micromolar ranges (>10 nM).

### 3.2. Chemiresistive Sensors Based on Conducting Polymers

Conducting polymers are of great interest in sensing due to their properties like mechanical flexibility, simple synthesis process, and good conductivity. One of the earliest H_2_O_2_ sensors based on a conducting polymer was constructed using polypyrrole due to its biocompatibility, easy processing and fabrication, and significant effect on conductivity in the presence of redox dopants [48]. The electropolymerized polypyrrole film exhibited a conductivity change in presence of H_2_O_2_ due to the introduction of additional holes in the film. The study compares the sensor performance of two types of films: one with GOD encapsulated on polypyrrole film and other with GOD encapsulated in the polypyrrole-MWCNT film to detect glucose. The sensor was found to measure H_2_O_2_ over a concentration range of 0–20 mM with a sensitivity of 0.9 mS cm^−1^ mM^−1^. The sensitivity of the sensor was enhanced to 2.6 mS cm^−1^ mM^−1^ (~3 times) by introducing MWCNTs with polypyrrole as the active thin film. However, the sensor was also found to be sensitive to environmental parameters like mechanical stress, temperature, and oxygen concentration. This study did not investigate the effect of interferent species and real samples on the sensor performance.

Polyaniline is another conducting polymer that has been used for H_2_O_2_ measurement [20,51,52] and subsequently used the sensor for glucose detection [50]. The conductivity of polyaniline strongly depends on the pH value of the solution and this property is used in H_2_O_2_ detection [20]. A polyaniline nanowire network was used for sensor preparation. Nanowires were used due to their superior sensing performance like rapid response time, higher sensitivity, and improved LOD due to increased surface area, and high porosity [50]. The nanowires were modified with silver nanoparticles (AgNPs) which catalyze the H_2_O_2_ reaction to generate hydroxy ions as a by-product that increases the pH in the vicinity [20]. The introduction of AgNPs improved the sensor response from 3% (no AgNPs) decrease in conduction current to ~20% (with AgNPs), when both sensors were exposed to 20 mM H_2_O_2_. The increased H_2_O_2_ concentration results in increase in pH which leads to decrease in conductivity of the polyaniline film. The measuring range of the sensor was 5–40 mM of H_2_O_2_. Moreover, the sensor can be used multiple times by regenerating the sensor surface using an electrochemical method [53]. Sensors were stable during 36 h testing.

The same group demonstrated a conducting polymer based multiple analyte detection platform. The sensing platform was used to test three physiological relevant analytes: H_2_O_2_, dopamine and ascorbic acid within a range of 1–10 mM for all three analytes [52]. The response time of the sensors was around 2 min. The sensor panel provides a generic platform which can be applied to measure the required analyte by changing the surface coatings. Further, the group reported a low-cost method of fabricating the sensor using inkjet printing [51]. The process was validated by fabricating a H_2_O_2_ sensor using polyaniline nanowires and Ag NPs. The fabricated sensor exhibited a measuring range of 1–20 mM with a response time of 3 min. The minimum printing resolution was around 200 μm which can be used to fabricate chemiresistors.

**Table 1:** A list of H_2_O_2_ chemiresistive sensors with the crucial sensor properties including LOD, measuring range, voltage bias, response time, buffer and working pH. All units are as mentioned in the top row unless specified. Where NR: Not reported, PPy: Polypyrrole, PANI: Polyaniline, Pt NPs: Platinum nanoparticles, SnO_2_: Tin oxide, Au: Gold.

Substrate	Target Analyte	Ligand/ Enzyme	LOD (mM)	Measuring Range (mM)	Voltage Bias (mV)	Response Time (s)	Buffer/ Working pH	Comments	Interference Tested	Ref
***Carbon nanotube based***										
PPy-MWCNT	H_2_O_2_/ Glucose	Dodecyl benzene sulfonate	NR	0–20	1	NR	NR	Investigated the sensitivity of temperature humidity etc.	No	[48]
CNT	Glucose	EGCG-GOD	8.7 nM	10 nM–1 μM	100	<400 (est.)	Working pH 7.4 Buffer: PBS	Sensor responds to all reactive oxidative species	Yes	[49]
SWCNT-PVP	Glucose	GOD	0.08	0.02–2	100	3	Working pH 5.5 Buffer: Acetate	Tested in juice & iced tea Stable for 5 consecutive tests	Yes	[19]
***Conducting polymer based***										
Au-PANI nanowires	H_2_O_2_	AgNPs	5	5–40	20	25	Working pH 5 Buffer: Phosphate (200 mM)	Stable response for 36 h Reusable sensor	Yes	[20]
MWCNT-PANI nanowires	H_2_O_2_	AgNPs	1	1–20	NR	180	NR	Inkjet printed sensors	No	[51]
MWCNT-PANI nanowire	H_2_O_2_ Glucose	PtNPs	2	2–10	500	240	NR	Inkjet printed	No	[50]
***Others***										
Alumina	Glucose	SnO_2_-GOD	0.5 (est.)	0.5–20	NR	50	Working pH 7.2 Buffer: Phosphate	Sensor sensitivity increases with deposition temperature of SnO_2_	No	[54]

Later, Platinum nanoparticles were used with immobilized GOD to fabricate a chemiresistive sensor for glucose detection [50]. The sensor was printed using an inkjet printer described in their previous work [51]. GOD catalyzes the conversion of glucose to gluconic acid and H_2_O_2_. The generated H_2_O_2_ is converted to hydroxide ions which is catalyzed by the platinum nanoparticles. The produced hydroxide ions resulted in a local pH change which subsequently changes the conductivity of the PANI nanowire layer. The PANI nanowire layer showed good reproducibility with a standard deviation of 2.7% (n = 5). The authors demonstrated a low-cost glucose sensor with a simple fabrication process with a linear measuring range of 2–10 mM with a LOD of 2 mM. The sensor measuring range (2–10 mM) was wider as compared to the SWCNT-PVP sensor reported (0.02–2 mM) [19]. However, the study does not comment on the effect on sensor response due to interfering species and physiological matrices like blood, urine, etc.

### 3.3. Chemiresistive Sensors Based on Other Materials

Metal oxides have been used to measure H_2_O_2_ using a chemiresistive geometry. For instance, a nanostructured SnO_2_ film on an alumina substrate along with immobilized GOD has been used to sense H_2_O_2_ and through that measure glucose [54]. The sensitivity of the SnO_2_ layer is due to adsorption of molecular oxygen (Reaction 3) and its reduction through the extraction of electrons from the conduction band of the SnO_2_ film.

(3) $$O_{2}+2e^{-}\;\to 2O_{\mathrm{ads}}^{-}$$

The electron extraction increases the potential barrier which in turn leads to a decrease in conduction. Upon exposure to GOD, glucose is converted to gluconic acid and H_2_O_2_, the gluconic acid converts to D-gluconate and H^+^. This H^+^ and H_2_O_2_ react with the $O_{\mathrm{ads}}^{-}$ to release the electron extracted from the conduction band of the tin oxide film, resulting in an increase in resistance. A suitable catalyst can further improve the sensor performance. A linear range was reported from 0.5 to 20 mM with the highest sensitivity when the tin oxide film was grown at 450 °C.

## 4. Conductometric Sensors

**Figure 6:**
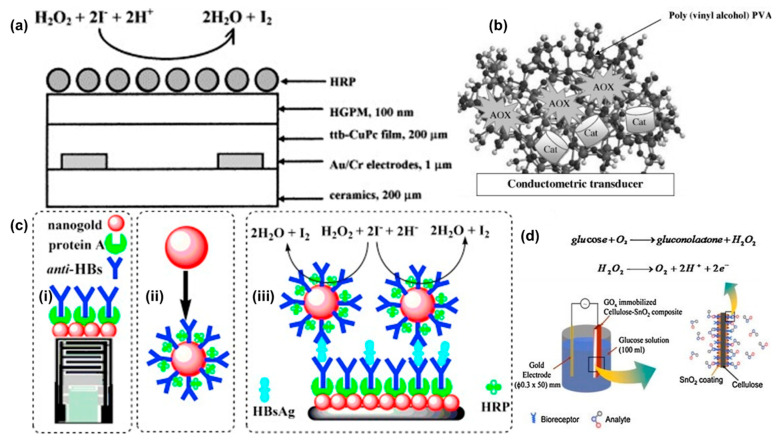
(**a**) A HRP conductometric sensor for enzymatic detection of H_2_O_2_. The sensor was fabricated using HRP as the enzyme and ttb-CUPc as the active/sensitive film to detect the released iodine molecule. Reprinted from [38] with permission from Elsevier. (**b**) A bi-enzyme sensor (Alcohol oxidase and catalase) to detect alcohol using conductometric transducer. Reprinted from [39] with permission from Elsevier. (**c**) (**i**) Schematics of a conductometric transducer working as an immunosensor (**ii**) A nanogold particle coated with anti-HBs and protein A (**iii**) Schematics of the sandwich immunoassay with the reactions involved to measure Hepatitis-B surface antigens. Reprinted from [42] with permission from Elsevier. (**d**) Schematics of a GOD immobilized cellulose-SnO_2_ composite electrode. Reprinted from [46] with permission from Elsevier.

One of the earliest conductometric sensors for H_2_O_2_ was developed in 1999 [43]. The sensor was constructed using tetra-tert-butyl copper phthalocyanine (ttb-CuPc) coated gold interdigitated electrodes on a ceramic substrate (Figure 6a). The phthalocyanine film was deposited using the Langmuir-Blodgett method. The sensing of H_2_O_2_ was based on oxidation of iodide ions by H_2_O_2_ in the presence of HRP (Reaction 1). The iodine concentration was measured by a conductometric sensor based on ttb-CuPc as the sensing layer. The effect of other interfering species in the sample was suppressed using a hydrophobic gas permeable membrane. HRP was immobilized on top of the gas permeable membrane. The sensor attained a steady state response in ~10 min which was due to slow conductivity changes in the phthalocyanine film. The highest sensor response was obtained within a pH range of 5.0–6.5. The measuring range was 0.005 to 0.3 mM with a sensitivity of 0.042 μS/μM of H_2_O_2_. The sensor worked continuously for 7 h with more than 30 measurements and had a storage stability of 90 days when stored at 4 °C.

Since then, multiple conductometric sensors have been reported in the literature. These sensors can be classified based on electrode material used for the sensors such as metal [43,55,56,57], metal nanoparticles [21,22,23,58], and others [59]. The sensors are compared based on crucial parameters like sensitivity, measuring range, LOD, potential bias, and response time. A summary of conductometric sensors is given in Table 2.

### 4.1. Conductometric Sensors Based on Metal Electrodes

Similar to the first H_2_O_2_ conductometric sensor, many sensors were prepared using interdigitated gold electrodes. One such sensor was prepared using a polyvinyl alcohol membrane to immobilize catalase [56], an enzyme that converts H_2_O_2_ to water. The measurement was done using an AC bias of 10 mV and 100 kHz frequency. The electrodes exhibited a linear range wider than the phthalocyanine sensor [43] from 0–100 mM H_2_O_2_ with a detection limit of 6 µM. The sensitivity of the sensor was 1 μS/μM of H_2_O_2_ which was around 20 times higher than the phthalocyanine H_2_O_2_ conductometric sensor (0.042 μS/μM) [43]. The response time of the sensor was less than 5 min, which was half of the previous sensor (~10 min) [43]. Interestingly, the sensor was shown to respond to cyanide as an interfering species due to the inhibitory effect of cyanide on the catalase enzyme.

Similar to glucose sensors, alcohols were also detected using alcohol oxidase enzyme (Reaction 4). The alcohol concentration can be correlated to either the decrease in oxygen or increase in hydrogen peroxide concentration. A dual enzyme sensor based on alcohol oxidase (AOX) and catalase was used to detect lower aliphatic alcohols including methanol, ethanol, and n-propanol [55]. The proposed dual enzyme system is one of the first such studies reported to measure the alcohol concentrations (Figure 6b). The generated H_2_O_2_ can be used by the catalase to convert it to oxygen and water (Reactions 4–6).

(4) $${\mathrm{RCH}}_{2}\mathrm{OH}+O_{2}\overset{\mathrm{AOX}}{\to }\;\mathrm{RCHO}+H_{2}O_{2}$$

(5) $$2H_{2}O_{2}\;\overset{\mathrm{Catalase}}{\to }{\;O}_{2}+H_{2}O\;$$

(6) $${\mathrm{CH}}_{3}{\mathrm{CH}}_{2}\mathrm{OH}+H_{2}O_{2}\overset{\mathrm{Catalase}}{\to }{\;\mathrm{CH}}_{3}\mathrm{CHO}+2H_{2}O$$

To fabricate the sensor, enzymes were immobilized on a polyvinyl alcohol photopolymerized network. The sensor showed a linear range for the three test alcohols: methanol, ethanol, and n-propanol up to 0.075 mM, 0.070 mM and 0.065 mM, respectively. In addition, an increase in the number of carbons in the alcohols resulted in a higher LOD with 0.5 µM for methanol, 1 µM for ethanol and 3 µM for n-propanol. The sensitivities of the sensors were 0.394, 0.363 and 0.317 μS/μM of methanol, ethanol and n-propanol which was around one order higher than the phthalocyanine H_2_O_2_ conductometric sensor (0.042 μS/μM) [43]. Sensors were stable for 3–4 months when stored in phosphate buffer at 4 °C. The sensor response was stable for three months with no significant changes when used 2–3 times a week. However, a 5% reduction in sensor response was observed at the end of the 4th month. There was no significant interference from compounds like lactic acid, ascorbic acid, oxalic acid, malic acid, glucose, tartaric acid, and citric acid.

For immobilized enzyme conductometric electrodes, the sample matrix can have a significant influence on the sensor response. Therefore, a second electrode which lacks enzyme coating was used to normalize the influence of the matrix. For example, a sensor with two electrodes was proposed to detect lactate in dairy products [57]. The sensing system consists of two pair of interdigitated gold electrodes fabricated on a ceramic substrate (Al_2_O_3_). The working electrode was coated with lactate oxidase and HRP while the second electrode was coated with a non-reactive bovine serum albumin layer. All measurements were performed at 10 mV AC bias with 100 kHz frequency in phosphate buffer at pH 6. The sensitivity of 5.58 μS/μM of lactate was two orders of magnitude higher than that of the first H_2_O_2_ conductometric sensor (0.042 μS/μM) [43]. The sensor showed a low detection limit of 0.05 µM. The biosensor was stable for up to five weeks when stored at 4 °C in a phosphate buffer solution and used intermittently. The sensor was tested with common interferents like glucose, fructose, and ascorbic acid. Apart from ascorbic acid, none of the compounds had a significant effect on the sensor’s response. The sensor was also tested in modified yogurt.

### 4.2. Conductometric Sensors Based on Metal Nanoparticles

Conductometric sensors have been fabricated using mainly two types of nanoparticles: gold [22,23,58], and platinum [21]. Nanoparticles provides a high surface to volume ratio resulting in an increase in enzyme loading which in turn could led to increased sensitivity of the sensor. One such study used both gold and magnetic nanoparticles to detect glucose [23]. Planar interdigitated electrodes were used to evaluate the effect of nanoparticles on the analytical performance of the glucose conductometric sensors. The measurements were performed using a 10 mV and 100 kHz frequency signal in phosphate buffer at pH 7.3. The gold nanoparticles functionalization increased the sensitivity of the sensor from 31 µS/mM (without) to 45 µS/mM (with). The LOD was also decreased from 50 µM (without) to 9 µM (with nanoparticles). The linear range for both with and without gold nanoparticles was 0.04 to 1 mM. The study further explores the sensor response with magnetic nanoparticles (Carboxy-Adambeads). The sensitivity was enhanced to 75 µS/mM of glucose concentration with a LOD of 3 µM. The study failed to provide a valid explanation for increased sensitivity with magnetic nanoparticles as compared to gold nanoparticles. Gold nanoparticles were also used in combination with chitosan to immobilize HRP for H_2_O_2_ detection [22]. The sensor uses chitosan to immobilize HRP due to its excellent mechanical strength,

Biocompatibility, non-toxicity, high permeability, and superior film forming ability. The sensor exhibited a linear response from 0–15 mM of H_2_O_2_ concentration. However, this sensor used high potential from 0–6 V for measurements which might not be ideal for sensing biomolecules.

Nanoparticles were also used to fabricate conductometric immunosensors for the detection of H_2_O_2_ using the H_2_O_2_-KI reaction (Reaction 1). A HRP-AuNP-anti-hepatitis antibody (HAb) based sensor was proposed for detection of Hepatitis surface antigen B, a major index for Hepatitis B [58]. The interdigitated electrode was coated with AuNPs and protein A followed by anti-HAbs. The measuring solution contained double codified AuNPs, Hepatitis B surface antigens, H_2_O_2_, and KI (Figure 6c). The addition of double codified AuNPs enhanced the sensor response. The sensor without AuNPs exhibited a linear range from 1.5–450 ng/mL which was increased to 0.1–600 ng/mL with AuNPs. The detection limit of the sensor was also improved to 0.01 ng/mL (with AuNPs) from 0.5 ng/mL. A 40% increase in sensitivity was observed with the addition of the AuNPs. The sensors showed good intra (4.9%) and inter (7.1%) reproducibility values at 1.5 ng/mL. The conductometric assay was validated using 40 serum samples against ELISA. All measurements were performed at a low potential bias of 10 mV at 100 kHz.

Another study used graphitic carbon nitrite (g-C_3_N_4_) and platinum NPs for H_2_O_2_ detection. Both g-C_3_N_4_ and platinum NPs (PtNPs) were used in multiple sensing research studies due to their peroxidase activity [21]. In this study, g-C_3_N_4_ nanosheets were functionalized with PtNPs to develop a conductometric immunosensor using the H_2_O_2_-iodide system. The nanosheets mimic a natural enzyme and reduce H_2_O_2_ in presence of iodide. Due to the low conductivity of the antigen-antibody complex, the immunoreaction causes a change in the conductivity. The PtNPs-g-C_3_N_4_ sensor exhibited a linear response from 0–50 µM of H_2_O_2_ where the sensor took around 5–6 min to achieve the steady state response. The peroxidase activity of the PtNPs-g-C_3_N_4_ network was achieved at 6.5 which can be because of H_2_O_2_ adsorption at lower/higher pH values. The immunoassay can detect AFP within 1.5 h which is faster than the conventional ELISA kits (~3 h). The assay also showed a linear relationship from 0.1 to 100 ng/mL for AFP with a lower LOD which is two orders of magnitude lower than the threshold for the human serum (10 ng/mL). Here the measurements were also done at 10 mV at 100 kHz similar to the previous study [58]. The sensor indicated an intra batch reproducibility with a coefficient of variance (CV) of 8.7% for 1 ng/mL and inter batch reproducibility with a CV of 10.9% for 1 ng/mL AFP.

Nanoparticles enhanced the sensitivity of the conductometric sensor by approximately 6 times as compared to the best sensitivity reported for the above described metal electrodes (5.58 μS/μM) [57]. Addition of nanoparticles facilitate a versatile platform to immobilize different protein to perform various immunoassays [21,58]. Four out of eight [21,55,57,58] studies discussed here have reported the interference test and tested the sensors with real samples.

### 4.3. Conductometric Sensors Using Other Materials

SnO_2_ with a band gap of 3.2 eV (room temperature) is widely used in gas sensors. The SnO_2_ layer is sensitive to H_2_O_2_ through adsorption of molecular oxygen (Reaction 3). The detailed sensing mechanism of SnO_2_ electrodes is described in Section 3.3. Here, a low-cost flexible and one-time use sensor was prepared using cellulose and SnO_2_ for glucose detection [59] (Figure 6d). The regenerated cellulose substrate was spin coated onto a silicon substrate and cured. SnO_2_ was deposited on top of the regenerated cellulose film by leaving the substrate immersed in the SnO_2_ solution for a given time. An increase in immersion time results in an increase in current which may be due to an increase in the amount of SnO_2_ crystals grown over time, thus improving the crystallinity of the grown layer. GOD was immobilized by physisorption when the cellulose-SnO_2_ substrate was left in the glucose solution for 16 h. The fabricated sensor exhibited a linear response from 0.5–12 mM of glucose concentrations when measurements were performed in phosphate buffer at pH 7.2. The sensor offers a low-cost alternative to enzyme based H_2_O_2_ sensors. However, it has a limited measuring range and required a high potential (up to 3V) for detection, which could lead to interference with other coexisting compounds present in the analyte.

## 5. FET Sensors

H_2_O_2_ is not an ideal molecule to detect using electrochemical reactions due to its slow reaction kinetics. Amperometric sensors overcome this by applying a suitable potential to accelerate the reaction kinetics. However, this overpotential limits the detection at low concentrations because of a high signal to noise ratio. Similar detection limit issues exist for potentiometric sensors. In FET sensors, the phenomenon of surface charging at the gate and the effect of it on the inversion layer created and the conductivity of the channel is non-linear and hence the amplification can result. FET sensors can be classified in to four major groups based on the active material used for the detection: silicon nitride [40,41,42,45,46,60,61,62], conducting polymers [63,64,65,66,67], metal oxide [44,68,69,70,71,72,73,74], and carbon nanomaterials [24,25,75,76,77,78,79]. The sensors are evaluated based on crucial parameters like measuring range, sensitivity, detection limit, and response time. FET sensors are summarized in Table 3.

### 5.1. FET Sensors Based on Silicon Nitride

Silicon nitride (Si_3_N_4_) has been widely used as a pH sensitive material for FETs due to its wide range of pH detection (pH 2 to 10) and good sensitivity (52–54 mV/pH). The pH response of the Si_3_N_4_ film coupled with enzymes like HRP that induce pH changes in the presence of H_2_O_2_ have been used in sensing. For instance, HRP [41,60] and electrochemical reduction using platinum electrodes [46] was used for direct H_2_O_2_ measurements. In contrast, GOD [40,42,45], invertase, and mutarotase [42] were used for detection of glucose and sucrose by converting these compounds into H_2_O_2_ and electrochemically reducing the H_2_O_2_ to generate protons for detection by the Si_3_N_4_ film.

The earliest FET H_2_O_2_ sensor was constructed by immobilizing HRP on a Si_3_N_4_ film, and a reference FET was also fabricated without any enzyme in the immobilized layer. The reduction of H_2_O_2_ using HRP is a three-step reaction. The first step involves the reduction of H_2_O_2_ while HRP is simultaneously oxidized. A mediator is used to convert the oxidized HRP back to its reduced form [41,75]. The working mechanism of HRP enzyme is shown below:

(7) $$H_{2}O_{2}+\mathrm{HRP}\left(\mathrm{red}\right)\to \mathrm{HRP}\;\left(\mathrm{oxd}\right)+2H_{2}O\;$$

(8) $$\mathrm{HRP}\;\left(\mathrm{oxd}\right)+M+{\;H}^{+}\to {\mathrm{HRP}}^{*}+{\;M}^{+}$$

(9) $${\mathrm{HRP}}^{*}+M+{\;H}^{+}\to \mathrm{HRP}\;\left(\mathrm{red}\right)+M^{+}$$

HRP based sensors used reducing agents like potassium iodide [41], potassium hexacyanoferrate (II) [41] and ascorbic acid [60]. In 1994, a study evaluated the sensitivity of the Enzyme FET (ENFET) using two reducing substrates: potassium hexacyanoferrate (II) and potassium iodide (KI) [41]. An increase in linear range was observed with increase in the concentration of reducing agents. With KI, the sensor exhibited a better sensitivity, but the measuring range was narrower in case of hexacyanoferrate. The reusable biosensor showed an activity loss of >10% after 1000 measurements. The detection range of the sensor was 5 μM to 2 mM at pH 6 with a LOD at 5 μM and a sensitivity of 15 mV/mM. The sensor had a response time of 30–90 s.

H_2_O_2_ ISFETs have been coupled with GOD to detect glucose (Reactions 10 and 11) [40,42,45,46,61,62]. Conventionally, these ISFETs used to target the protons generated from the dissociation of gluconic acid in solution (Reaction 11). However, the sensitivity of the sensors was limited by the low dissociation constant (pKa~3.8) of gluconic acid [80]. To increase the sensitivity, the researchers used the generated H_2_O_2_ to produce two additional hydrogen ions (Reaction 12). This facilitates the increase in sensitivity of the ISFET. In 1996, the first study demonstrated the feasibility of using H_2_O_2_ electrolysis to increase the sensitivity of a glucose ISFET [40].

(10) $$C_{6}H_{12}O_{6}+H_{2}O+O_{2}\;\overset{\mathrm{GOD}}{\to }{\;C}_{6}H_{12}O_{7}+H_{2}O_{2}$$

(11) $$C_{6}H_{12}O_{7}\;↔C_{6}H_{11}O_{7}{}^{-}+{\;H}^{+}$$

(12) $$H_{2}O_{2}\to 2H^{+}+O_{2}+2e^{-}$$

Apart from the analyte present, the availability of oxygen in the solution also determines the response as seen from reaction 10. The regeneration of oxygen by decomposition of the generated H_2_O_2_ (Reaction 12) through electrochemical means can serve to enhance the signal obtained from the catalysis of glucose by GOD (Reaction 10). The additional oxygen would also facilitate glucose measurement over a wider range. The electrolysis of H_2_O_2_ was facilitated using a platinum electrode and applying a 0.65 V bias to the electrode. This method also established the baseline of the sensor which is critical for reliable measurements using FETs due to the high inherent drift rates (0.1–1 mV/h). In conventional methods, the baseline was established by introducing a glucose free solution before the measurement. However, this method established the baseline by removing the potential bias which alleviates the need of a glucose free solution.

With no potential bias, the upper limit of measurement was 1.5 mM which is due to limited oxygen availability for reaction 11. With potential bias, the upper limit was increased to 5 mM from 1.5 mM (no potential bias) which is due to additional oxygen generated by reaction 12. The sensitivity of the sensor was ~40 mV/mM in a 5 mM phosphate buffer and 17 mV/mM in a 20 mM phosphate buffer which is similar to the first sensor (~15 mV/mM). The reduction in sensitivity was observed, when the buffer strength is increased from 5 mM to 20 mM due to the facilitated diffusion of protons from the enzyme layer to the bulk at higher buffer concentrations. Further, the response time of the sensor was around 8 min which was higher than the first sensor (<1.5 min) [41].

The ISFET glucose sensor still had a slower response time which was dealt in a later study by reducing the thickness of the enzyme layer on top of the gate [42]. The sensor was coated with multiple enzymes (invertase and mutarotase) to detect sucrose. Invertase breaks down the sucrose molecule to α-D-glucose and fructose (Reaction 13). α-D-glucose was converted to β-D-glucose using mutarotase (Reaction 14), and then the detection was done using a glucose ISFET sensor (Reactions 10–12).

(13) $$\mathrm{Sucrose}\;\overset{\mathrm{Invertase}}{\to }\;\alpha -D-\mathrm{glucose}+\mathrm{fructose}\;$$

(14) $$\alpha -D-\mathrm{glucose}\;\overset{\mathrm{Mutarotase}}{\to }\;\beta -D-\mathrm{glucose}$$

**Figure 7:**
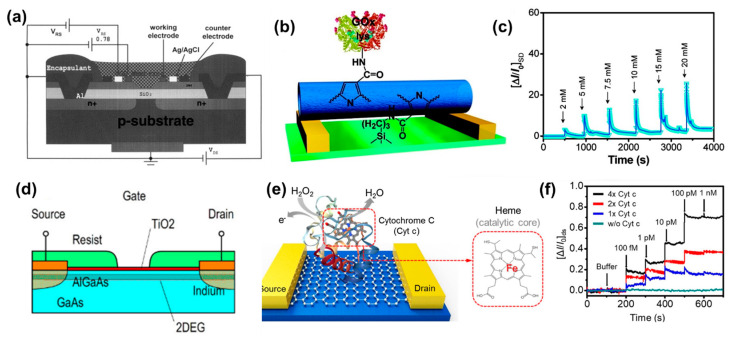
(**a**) A solid ISFET glucose sensor with a ladder-type platinum electrode as the working electrode, a counter electrode and an Ag/AgCl reference electrode. Reprinted from [75] with permission from Elsevier. (**b**) GOD-Carboxylated Polypyrrole nanotubes FET sensor for detection of H_2_O_2_ and glucose. Reprinted (adapted) with permission from [57]. Copyright 2008 American Chemical Society. (**c**) Real time current response due to of the sensor described in Figure 7b when it is exposed to H_2_O_2_ ranging from 2–20 mM. Reprinted (adapted) with permission from [57]. Copyright 2008 American Chemical Society. (**d**) A TiO_2_ thin film FET to monitor the cell viability with H_2_O_2_ as the marker. Reprinted with permission from [64]. (**e**) Schematics of a cytochrome-c (cyt c) based ultrasensitive H_2_O_2_ sensor. Reprinted from [73] with permission from Elsevier. (**f**) Plot showing the stepped current response, when the sensor is exposed to various concentration of H_2_O_2_ (0.1 pM to 1 nM), while different color solid line represents the cytochrome-c loading on the sensor no cyt c(green), 1× cyt c(blue), 2× cyt c(red) and 4× cyt c (black). Reprinted from [73] with permission from Elsevier.

A thin photopolymer (Polyvinyl alcohol-Styrylpyridinium) membrane was spin coated on top of the Si_3_N_4_ layer. The glucose ISFET exhibited a linear range from 1.67–16.67 mM with a response time of less than 5 min. The platinum electrode also had a significant effect on the sensitivity of the sensor. With increasing platinum electrode area, the sensitivity of the ISFET increases due to an increase in the number of protons generated in the vicinity of the pH-gate. However, a detailed study of the platinum electrode area with respect to the area of the gate needs to be done before establishing any direct relationship between the two factors. A similar ENFET was proposed to detect glucose using platinum electrodes on top of the gate of the ISFET [45,81]. The study proposed a ladder like platinum electrode designed to further improve the sensitivity and measuring range of the sensor (Figure 7a). The polarization potential was also investigated by a later study using cyclic voltammetry [46]. The GOD and lactate oxidase were immobilized on the sensor for detection of glucose and lactate, respectively. Si_3_N_4_ has advantages like good pH sensitivity, a wide pH working range and a simple manufacturing process which needs one additional deposition step for device fabrication.

### 5.2. FET Sensors Based on Conducting Polymers

Conducting polymers are used to fabricate sensors due to their ability to readily undergo oxidation and reduction, simple polymerization, reproducible deposition, and their stability in aqueous solutions. Common conducting polymers used in FET sensors are polyaniline [61,75,76,82], polypyrrole [65,66,67] and poly(3,4ethylenedioxythiophene) (PEDOT) [63,64,83,84,85,86].

#### 5.2.1. Polyaniline

Some of the initial conducting polymer based H_2_O_2_ sensors were constructed using polyaniline [61,75,76] due to its two distinctive properties. Firstly, the polymer has two conducting/insulating transitions induced by electrochemical oxidation: leucoemeraldine (insulating) to emeraldine (conducting); further oxidation of emeraldine to pernigraniline (insulating). Second, only the protonated form of emeraldine is conductive. Therefore, the conductivity of polyaniline can be changed either by changing the pH of the solution or in the presence of a redox species in the solution [87,88]. One example was a sensor using HRP as enzyme that catalyzes the conversion of H_2_O_2_ (detailed reactions in Section 5.1). This study was the first to demonstrate the use of a HRP-polyaniline based enzyme switch to measure H_2_O_2_ [75]. Polyaniline was grown on carbon electrodes with silver paint as the electrical contacts. In the study, polyaniline acts as the electron donor without any external mediator for the electron transfer. It was proven that the response is due to the oxidation of the polyaniline instead of other changes such as pH, which are typical in enzyme catalyzed reactions. The response of the device can be measured using both switching time and switching ratio. The device was calibrated using a single point calibration method to measure unknown concentrations. The devices measured the H_2_O_2_ concentration with an error of 0.03 mM within a 95% confidence interval. However, the device can only measure H_2_O_2_ concentrations below 0.5 mM. This was because the sensor response was inhibited at higher H_2_O_2_ due to formation of oxyperoxidase, a relatively stable form of the HRP enzyme. The sensor showed a response time of 100 s and a LOD of ~0.2 mM. The polyaniline film was reset electrochemically by reducing the film. However, the residual oxyperoxidase form of HRP posed a serious problem for repeatable use.

Polyaniline films are also doped by anions like bisulphates and chlorides. At pH > 5, the deprotonation of the emeraldine form results in an insulating polyaniline layer which limits its application in physiological settings. The deprotonation is accompanied by the removal of anions like bisulphates and chlorides [89]. These small anions can be substituted with long chain polymeric anions like poly (aniline-co-N-propane sulfonic acid aniline) (PSPANI) [76] or poly (acrylic acid) (PAA) [61] to prevent the deprotonation of polyaniline at neutral pH. Raffa et al. modified polyaniline to prepare poly(aniline-co-N-propane sulfonic acid aniline) (PSPANI) [76]. The polymer exhibited a good conductivity at pH 7. The sensor was prepared by modifying PSPANI with HRP to detect H_2_O_2_. The FET worked within a range of 0.025–1 mM with a response time of 100–300 s. The switching rate of the sensors was 0.126 μA/s. Another study used PAA to maintain the polyaniline conductivity at near neutral pH [61]. Here, the polyaniline was modified during the electropolymerization to generate a hybrid film. GOD was immobilized on the PANI-PAA film for specific detection of glucose within a range from 0–9 mM which was a significant improvement from the previous polyaniline H_2_O_2_ FET sensors. The sensor had a rapid response time of <1 s with a sensitivity of 1 nA/mM. Although multiple FET sensors are constructed using polyaniline, they have a few limitations, such as conductivity degradation at elevated pH conditions, hysteresis, and limited shelf life. Further details are discussed in this review [90].

#### 5.2.2. Polypyrrole

Polypyrrole is popular as a sensing material due to its good conductivity (10–100 S/cm), temperature stability, and ambient air storage stability with no significant changes in conductivity for at least 2 weeks [91]. Polypyrrole nanotubes have been used for detection of H_2_O_2_ as well as other analytes like glucose [65,66,67]. For example, a study reported a liquid gated FET sensor based on GOD functionalized polypyrrole nanotubes [65]. Carboxylated polypyrrole nanotubes enabled high enzyme loading on the sensor surface due to their high surface area and high density of surface functionalization groups (Figure 7b). Upon glucose addition, the current increased due to H_2_O_2_ generated from the reaction of glucose with GOD which changes the charge transfer characteristics of the polymer. Then, it gradually decreased due to deprotonation of the conducting polymer by the positive gate potential (Figure 7c). The sensor showed a ~3.75% current change per mM of glucose concentration relative to the baseline current when the sensor was not exposed to glucose. The measuring range was from 2–20 mM with 0.5 mM as the LOD. The sensor response time was 5–10 s. A different liquid gated FET was fabricated using a reduced graphene oxide(rGO) and polypyrrole nanotube composite [66]. The nanocomposite was created using the electrostatic interaction between positively charged polypyrrole nanotubes and negatively charged rGO. Individually, both rGO and nanotubes were sensitive to H_2_O_2_ concentration. Moreover, the composite exhibited a better sensitivity due to increased surface area, strong interactions between rGO and polypyrrole nanotubes, and improved signal transduction attributed to enhanced semiconductor behavior of the composite. The sensor extended a LOD of 0.1 nM with a measuring range from 0.1 nM to 100 nM. The sensor showed a sensitivity of ~2% change/decade change of the H_2_O_2_ concentration with respect to the baseline. A fast response time, no significant interference from common interferents like ascorbic acid, uric acid, and glucose, and long-term storage stability (1 month in air) make the sensor a good candidate for real sample applications such as in the food industry or for environmental samples.

The rGO-polypyrrole nanotube composite sensor functionalized with GOD was also used to measure glucose concentrations in diluted human, bovine and horse serum samples in a subsequent study [67]. The sensor exhibited a wide measuring range from 1 nM to 100 mM with a detection limit of 1 nM. The sensor showed a better sensitivity of ~5% change/decade which was 2.5 times better than the previous sensor [66]. The enzyme functionalization does not have any influence on the response time (<1 s). The sensor also had the similar repeatability and storage stability (1 month in air).

#### 5.2.3. PEDOT

PEDOT is a positively charged polymer commonly doped with polystyrene sulfonate (PSS), a negatively charged polymer. PEDOT: PSS is used to fabricate sensors with excellent stability and a wide range of operating pH. Organic thin film transistors (OTFTs) have been fabricated using PEDOT: PSS as the conducting polymer due to its ability to form stable thin films with sufficient conductivity [63,64,83,84,85,86]. OTFTs rely on electrostatic gating based on electrochemical doping or de-doping of the conducting polymer film and electrolyte solution. The conductivity of these devices is modulated by gate voltage. The sensing mechanism of the device can be explained by two mechanisms: ion-leveraged [92] and electrochemical [93]. In the ion-leveraged mechanism, the positive charge present in the solution migrates to the PEDOT: PSS film and changes its conductivity by disrupting the tunneling of holes in the film. In the electrochemical mechanism, H_2_O_2_ gets oxidized at the gate electrode and the charge balance is maintained by the reduction of PEDOT in the conducting polymer film. Y^+^ represents the positively charged ions present in the solution (Reaction 15).

(15) $${\mathrm{PEDOT}}^{+}:{\mathrm{PSS}}^{-}+{\;Y}^{+}+e^{-}↔\mathrm{PEDOT}+Y^{+}:{\mathrm{PSS}}^{-}$$

These devices need low operating voltages, low-cost substrate, low processing temperatures, and have simple structures which makes them easy to manufacture and integrate in microfluidic chips. In one example of a PEDOT:PSS-GOD based OTFT, a gate voltage was applied to measure glucose concentrations in a buffered solution [83]. The change in glucose concentration was measured using the change in drain-source current. The GOD converted glucose to gluconic acid and hydrogen peroxide. The generated H_2_O_2_ is oxidized at the platinum gate electrode which results in a change in drain-source current. The sensor exhibited a narrow measuring range from 0.1–1 mM with a response time of ~1 min. Similar sensors were reported in other studies with improved response time and measuring range [63,64,84,85,86]. Another study has used GOD in a PEDOT:PSS matrix to form the channel of the OTFT [64]. The sensor reported a wide range of detection from 1.1 to 16.5 mM with a rapid response time of 20 s. The sensitivity of the sensor of 1.65 μA/mM of glucose concentration and the detection limit of ~1 mM makes it suitable for blood glucose measurements. The TFT sensor was further modified by covalently immobilizing the enzyme on the sensing layer using methacrylate polymer chains [86]. The sensor exhibited a good enzyme activity with a wide measuring range 0.01–100 mM for glucose and 0.01 mM as the detection limit. However, the response time of the sensor was ~360 s which was longer than for the previous sensors [64,83]. The sensor was stable for 100 days in storage with intermittent measurements. The detection range of the TFT sensors was extended to lower concentrations by using a TiO_2_ nanotube array and platinum nanoparticles as the gate electrode [85]. The sensor showed a wide measuring range from 0.001–5 mM for H_2_O_2_.

### 5.3. FET Sensors Based on Metal Oxides

The sensitivity of ENFETs can be improved by oxidizing H_2_O_2_ through an applied external potential bias. However, in order to apply a potential, it is necessary to add an additional electrode on top of the gate of the FET. Therefore, some studies have used metal oxides as the catalyst to circumvent the need for an additional electrode. Metal oxides such as manganese dioxide (MnO_2_) [62,68], iridium oxide [69], lanthanide perovskite oxide [44], titanium oxide (TiO_2_) [71], iron (III) oxide (Fe_2_O_3_) [72], and zinc oxide (ZnO) [74,94] were used to catalyze the oxidation of H_2_O_2_. One of the earliest metal oxides used was MnO_2_, which catalyzed the conversion of H_2_O_2_ into H_2_O and O_2_ through a sequence of steps (Reactions 16–19) [68,95].

(16) $${\mathrm{MnO}}_{2}+H_{2}O\;\to \mathrm{MnO}{\left(\mathrm{OH}\right)}_{2}$$

(17) $$\mathrm{MnO}{\left(\mathrm{OH}\right)}_{2}\to {\mathrm{MnO}}^{2+}+2{\mathrm{OH}}^{-}$$

(18) $${\mathrm{MnO}}^{2+}+H_{2}O_{2}+4{\mathrm{OH}}^{-}\to {\mathrm{MnO}}_{4}^{2-}+\;3H_{2}O$$

(19) $${\mathrm{MnO}}_{4}^{2-}+H_{2}O\to {\;\mathrm{MnO}}_{2}+0.5{\;O}_{2\;}+2{\mathrm{OH}}^{-}$$

The first study to use MnO_2_ as catalyst for H_2_O_2_ conversion utilized a H^+^ sensitive ENFET covered with a manganese dioxide (MnO_2_) doped Bovine serum albumin (BSA) membrane [68]. This outer membrane used MnO_2_ powder to catalyze the conversion of H_2_O_2_ to H_2_O and O_2_. The detection limit of the sensor was 2.7 mM with a response time of 12 min. The sensitivity of the sensor was estimated to ~2.35 mV/mM of glucose concentration within a measuring range up to 20 mM. The MnO_2_-BSA membrane facilitated the diffusion of extra oxygen required by GOD resulting in a wider linear dynamic range. The oxidizing ability of MnO_2_ strongly depends on the pH range and increases with an increase in pH. In contrast, the stability of GOD decreases with pH. Therefore, the sensor response was highest at pH 8.1 and it decreases at higher pH values. Another study further modified the ENFET using MnO_2_ nanoparticles to increase the dynamic range and reduce the response time. An increase in pH is observed when MnO_2_ nanoparticles were used instead of bulk MnO_2_ particles. Therefore, a new mechanism was proposed to explain the pH increase (Reactions 20–23) [62].

(20) $$2{\mathrm{MnO}}_{2}+H_{2}O_{2}\;\to 2\mathrm{MnOOH}+O_{2}$$

MnOOH can undergo disproportionation (Reaction 23) or reduction (Reaction 22).

(21) $$2\mathrm{MnOOH}+H_{2}O_{2}+4H^{+}\;\to 2{\mathrm{Mn}}^{2+}+4H_{2}O+O_{2}$$

(22) $$2\mathrm{MnOOH}+2H^{+}\;\to {\mathrm{MnO}}_{2}+{\;\mathrm{Mn}}^{2+}+2H_{2}O$$

(23) $$C_{6}H_{12}O_{6}+{\mathrm{MnO}}_{2}+H^{+}\;\overset{\mathrm{GOD}}{\to }{\;C}_{6}H_{12}O_{7}+H_{2}O+{\;\mathrm{Mn}}^{2+}$$

The sensor showed a wider range of detection from 0.025–1.9 mM and the response time was reduced to 140 s from ~12 min (for bulk MnO_2_ particles) [68]. The sensitivity of the sensor can be estimated as ~10 mV/mM. The nanoparticle sensor enhanced the sensitivity as compared to bulk oxide MnO_2_ particles (~2.35 mV/mM). The detection limit was also extended to the lower H_2_O_2_ concentrations but the upper detection was limited to 1.9 mM as opposed to 20 mM for bulk MnO_2_ particles [68].

In a later study, three different H_2_O_2_ sensitive materials: Ir(OH)_3_, Prussian blue and a polyvinyl pyridine membrane containing HRP-Osmium, were investigated as functional catalyst materials [69]. Iridium hydroxide was the most stable catalyst even though it has the lowest catalytic activity. However, it can be used over a broad pH range (3.5–9) for detection of high concentrations of H_2_O_2_ (0.1 mM to 0.1 M) with a sensitivity of 400 mV/decade of concentration change. Prussian blue has a medium catalytic activity and the lowest sensitivity (290 mV/decade) among all three tested materials. It can operate in a pH range of 4.5–6 for detection of H_2_O_2_ concentrations from 0.01 to 1 mM. HRP with Osmium as the mediator possesses the highest catalytic activity and highest sensitivity (700 mV/decade) but it is less stable compared to both of the more sensitive materials. Nevertheless, it can only operate in a narrow pH range of 6.5–7.5 for the detection of low concentrations of H_2_O_2_ (0.1 to 10 μM). Another study reported a lanthanide perovskite oxide based FET sensor where the perovskite worked as a catalyst for H_2_O_2_ decomposition due to the presence of oxygen vacancies [44]. Tantalum oxide was used as the gate electrode. The sensitivity of the sensor was 35 mV/decade within a measuring range of 0.005–0.2 mM. The response time of the sensor was around 30 min which was higher than the other metal oxide sensors [62,68] The detection limit of the sensor was around 4 μM which can be extended by changing the stoichiometry of the perovskite oxide.

Another study utilized a biocompatible thin film FET sensor for assessing the cell viability in the culture [71] through peroxide detection (Figure 7d). A TiO_2_ thin film was used due its biocompatibility and minimal cell adhesion [96]. The chances of cell survival are reduced with the increase in H_2_O_2_ concentration. So, it can serve as a good indicator for cell viability. The sensitivity of the FET was strongly dependent on the testing medium. A sensitivity of 4.5 mV/μM was observed in Dulbecco’s Modified Eagle medium while a 10 times reduction was observed in phosphate buffer (0.41 mV/μM). The response time of the sensor was 5 min.

Nanorods have also used to increase the signal to noise ratio and to enhance the sensitivity of the FET based sensors. ZnO nanorods were used to fabricate efficient electrodes for applications like device fabrication drug delivery, and others. Using ZnO nanorods, a multiplex FET was constructed to detect three analytes: glucose, cholesterol, and urea (urea sensor was not discussed here as it was not linked to H_2_O_2_) [74]. In the presence of respective oxidases, glucose and cholesterol generate H_2_O_2_ which produces changes to the charge transfer properties of the ZnO film resulting in the sensor signal. The sensor response was linear from 0.05–70 mM and 0.01–45 mM for glucose and cholesterol, respectively. The sensitivity of the sensor was 32.27 μA mM^−1^cm^−2^ and 17.1 μA mM^−1^cm^−2^. The sensor response was tested in real samples like mouse blood and serum. The serum sample measurements were similar to buffered solution measurements, but the sensor response diminished by around 7% and 19% for glucose and cholesterol respectively in mouse blood. The sensor showed a stable response for 40 days with multiple measurements. Similarly, a non-enzymatic sensor was also constructed using ZnO nanorods coupled with NiO quantum dots [94]. NiO reacts with glucose to generate H_2_O_2_ and then ZnO nanorods can detect the generated H_2_O_2_ as described above. The sensor exhibited two linear ranges: one from 0.001–10 mM with a sensitivity of 13.14 μA/mM and second from 10–50 mM with almost half the sensitivity (7.31 μA/mM).

Multiple metal oxide bulk and nanomaterials can be deposited on the gates of FETs for H_2_O_2_ detection. ZnO nanoparticles are becoming more popular due to their high specific surface area, and efficient enzyme immobilization leading to a high sensitivity. Further, ZnO nanorod FETs showed a good response within the clinically relevant range in real samples like serum or blood. Therefore, ZnO can be a potential candidate for clinical applications.

### 5.4. FET Sensors Based on Carbon Nanomaterials

Carbon nanomaterials are widely used for fabricating biosensors due to their excellent mechanical and electrical properties. Various types of carbon based nanomaterials have been used to construct FET based sensors, such as CNTs [72,97], graphene [24,77,78,79,98,99], and reduced graphene oxide (r-GO) [25,66,67,98]. Several reviews have also been published on FETs constructed from carbon nanomaterials [100,101,102].

CNT are used in various sensors due to their smaller size (generally 1 μm in length and 1–3 nm diameter), high surface sensitivity, chemical stability, good electrical conductivity, and high tensile strength [100]. However, the use of CNT in sensors faces challenges such as low dispersion in common solvents like water; and separation of semiconducting CNT from metallic CNT, which are good for sensing applications [103]. Recent studies have used dispersants like polymers [97], surfactants, and green tea [104]. Only a few examples exist where CNT sensors have been used in the detection of H_2_O_2_. A study reported the use of EGCG combined with CNT to fabricate a H_2_O_2_ sensitive FET sensor [72]. The measuring range of the sensor was from 10^−5^ to 10^−3^ M with a detection limit of <5 μM. The response time of the sensor was ~500 s.

Graphene, a single 2D sheet of carbon, has strengths such as high surface to volume ratio, and a flat surface resulting in easy and effective surface functionalization. Chemical vapor deposition (CVD) can be used to produce large graphene sheets which offers advantages like uniform binding and better control over the surface modifications with π-π interactions [98]. CVD grown graphene is also easier to integrate with contacts and finds use in sensors for detection of various analytes through the detection of H_2_O_2_. For example, a FET was fabricated using CVD grown graphene functionalized with GOD to detect glucose [77]. GOD was functionalized on the graphene film by pyrene butanoic acid succinimidyl ester. The linker used the pyrene group for π-π interaction with the graphene surface and an amide bond on the other end with GOD. The catalytic activity of GOD resulted in an increase in graphene layer conductance leading to the sensor response.

**Table 3:** Summary of various FETs used for H_2_O_2_ measurement where OC: operating conditions; PANI: Polyaniline; PAA: poly acrylic acid; pDAB: poly(1,2 diaminobenzene); HRP: Horseradish peroxidase; PVP: poly(4-vinylpyridine-co-styrene); MOSC: Metal oxide semiconductor capacitor; 3-aminopropyltriethoxysilane (APTES), PPyNT: Polypyrrole nanotube, Pt: Platinum, I_s_: source current, V_ds_: Drain potential with respect to source, V_g_: is gate voltage, I_ds_: current between drain and source, V_d_: Drain potential, V_bias_: potential bias applied between the Pt electrode and reference electrode, I _bias_: current bias, pDAB: poly(1,2-diaminoben-zene), ITO: Indium titanium oxide, GluD: Glutamate dehydrogenase, UA: Uric acid, AA: Ascorbic acid and MoS_2_: Molybdenum disulphide.

Substrate	Target Analyte	Ligand/ Enzyme	LOD (µM)	Sensitivity	Measuring Range (mM)	OC	Response Time (s)	Working pH & Buffer	Comments	Ref
***Silicon nitride FET***										
Si_3_N_4_-FET	H_2_O_2_	HRP	5	~15 mV/mM (est.)	<2	I_s_: 300 μA V_ds_: 2 V	30–90	Buffer: Phosphate (10 mM) Working pH: 6	<10% reduction in enzyme activity after 1000 measurements	[41]
Si_3_N_4_-FET/Pt electrode	Glucose	GOD	NR	~40 mV/mM	<5	V_bias_: 0.64 V	~480	Buffer: Phosphate (5–20 mM) Working pH: 7.4	Baseline established by removing the potential bias	[40]
Si_3_N_4_-FET/Pt electrode	Glucose & sucrose	GOD & Invertase-mutarotase-GOD	~50 (est)	NR	1.67–16.67	V_bias_: 0.7 V	180–300	Buffer: Phosphate (10 mM) Working pH: 7.4	Greater Pt area, increases sensitivity	[42]
Si_3_N_4_-FET/Pt electrode	Glucose	GOD	1000 (est.)	~11 mV/mM (est.)	1–10	V_bias_: 0.7 V	~60	Buffer: Phosphate (10 mM) Working pH: 7.4	Ladder shape Pt electrode was used for potential bias	[45]
Si_3_N_4_-FET	H_2_O_2_	Pt	10000	5 mV/mM	10–100	I_ds_: 0.1 mA V_ds_: IV	300	Buffer: Phosphate (100 mM) Working pH: 7.2	Used for glucose and lactate	[46]
***Conducting polymers***										
Carbon	H_2_O_2_	PANI-pDAB-HRP	100	NR	<0.5	V_g_: 200 mV V_d_: 20 mV	~100 s	Buffer: citrate-phosphate-Na_2_SO_4_ Working pH: 5	HRP inhibition at H_2_O_2_ concentration > 0.5 mM.	[75]
Kapton-Carbon	H_2_O_2_	PSPANI-HRP	25	0.126 μA/s	0.025–1	V_g_: 0 V V_d_: 20 mV	100–300 s	Buffer: HEPES-KNO_3_ (100 mM) Working pH: 7	Sultonation improves the PANI conductivity at pH 7	[76]
Si_3_N_4_-FET	Glucose	PANI-PAA-GOD	NR	1 nA/mM	0–9	V_g_: 20 mV V_ds_: 10 mV	<1 s	Buffer: McIlvaine Working pH: 5	PANI-PAA film was deposition by electropolymerization	[61]
PEDOT-TFT	H_2_O_2_ & Glucose	GOD	100	NR	0.1–1	V_d_: 0.2 V V_g_: 0–0.6 V	~60 s	Buffer: PBS Working pH: 7.14	pH independent response from pH 5 to 9	[83]
PEDOT-TFT	Glucose	GOD	1	0.1 V/decade	<1	V_ds_: −0.2 V	NR	Buffer: PBS (15 mM) Working pH: 6.8	Sensitivity can be improved by increasing Vg	[63]
PEDOT-TFT	Glucose	GOD	<1000	1.65 μA/mM	1.1 to 16.5	V_ds_: −1.5 V V_g_: 0.0 V	10–20 s	NR	Sensor was encapsulated in cellulose acetate membrane	[64]
Liquid gate-FET	H_2_O_2_ & Glucose	PPyNT-GOD	500 (est.)	3.75%/mM (est.)	2–20	V_ds_: −0.01 V V_g_: 0.01 V	5–10 s	Buffer: PBS (10 mM) Working pH: 7.0	High enzyme loading was achieved	[65]
TFT	H_2_O_2_ & Glucose	PEDOT-GOD	1	0.79–3 μA/mM	0.001–5	V_ds_: −0.4 V V_g_: 0.4 V	<20 s	Buffer: PBS Working pH: 7.4	Used as both optical and electrochemical	[84]
TFT	Glucose	PEDOT-GOD	10	NR	0.01–100	V_ds_: −0.7 V V_g_: 0.7 V	~360 s	Buffer: PBS (120 mM)	Stable for 100 days with covalently immobilized GOD	[86]
TFT	H_2_O_2_ & Glucose	PEDOT-TiO_2_-GOD	1	0.126%/decade	0.001–5	V_ds_: −0.1 V V_g_: 0.4 V	~1000 s (est.)	Buffer: PBS (10 mM) Working pH: 7.0	Stable for 10 days with intermittent testing	[85]
Liquid gate FET	H_2_O_2_	rGO-PPy NTs	0.1 nM	2%/decade	0.1–100 nM	V_g_: 0.1 V V_ds_: −0.01 V	<1 s	Buffer: PBS Working pH: 7.4	Stable up to 1 month, when stored in air	[66,67]
***Metal oxides***										
Glass-ITO-SnO_2_	Glucose	GOD-MnO_2_	2700	2.35 mV/mM	<20	No bias	720	Buffer: Phosphate-KOH (5 mM) Working pH: 8.1	Dynamic range strongly depends on pH value	[68]
Si_3_N_4_-FET	Glucose	GOD-MnO_2_ NPs	20	NR	0.025–1.9	No bias	~140 s	Buffer: Tris (10 mM) Working pH: 7.4	Repeatability: 1.9% (RSD) for 7 measurements	[62]
FET	H_2_O_2_	Iridium oxide	100	400 mV/dec	0.1–10	I_bias_: 25 nA	NR	Working pH: 3.5–9	-	[69]
		Prussian blue	10	290 mV/dec	0.01–1	I_bias_: 50 nA		Working pH: 4.5–6		
		Os-PVP-HRP	0.1	700 mV/dec	10^−7^–10^−5^ M	I_bias_: 25 nA		Working pH: 4.5–6		
Ta_2_O_5_-FET-Pt	H_2_O_2_	Perovskite oxide	4	35 mV/dec	0.005–0.2	I_bias_: 25 nA	1800	Buffer: Phosphate Working pH: 7	Change in stoichiometry of oxide can result in lower detection limit	[44]
FET	H_2_O_2_	TiO_2_	NR	4.5 mV/μM (DMEM media)	NR	I_ds_: 0.1 mA V_ds_: 1 V	300 (est.)	Buffer: Phosphate	DMEM media	[71]
FET	Glucose	ZnO-NiO quantum dots	26	13.14 μA mM^−1^(0.001–10 mM)	0.001–50	V_g_: 1.2–2 V V_ds_: 0.0 V	NR	Buffer: PBS (10 mM) Working pH: 7.4	Tested in whole blood and serum	[94]
Liquid gate FET	Glucose	ZnO rod-GOD	0.07	32.27 μA mM^−1^cm^−2^	0.05–70	V_g_: 0–2 V	NR	Buffer: PBS (50 mM) Working pH: 7.4	Mice blood, serum	[74]
	Cholesterol	ZnO rod-COD	0.04	17.1 μA mM^−1^cm^−2^	0.01–45	V_g_: 2–3 V	NR			
***Carbon nanomaterials***										
Graphene-FET	Glucose	GOD	100	~1 μA/mM (est.)	<10	V_ds_: 0.1 V V_g_: 0 V	<200 s (est.)	Buffer: PBS (10 mM) Working pH: 7.2	Glutamate was also detected using the sensor with GluD	[77]
OTFT	Glucose	Graphene-Chitosan-GOD	0.01	370 mV/dec	0.01–1 μM	V_g_: 0.4 V V_ds_: 0.05 V	~500 s	Buffer: PBS Working pH: 7.4	Investigated the effect of interference of UA and AA	[98]
Graphene-FET	Glucose	Silk fibroin-GOD	100	2.5 μA/mM	0.1–10	V_g_: 0 V V_ds_:0.1 V	~100 s	Buffer: PBS (10 mM) Working pH: 7.4	Stable for 10 months at room temperature	[79]
FET	H_2_O_2_ & Glucose	Graphene-Chitosan-PtNPs-GOD	0.03	91.7 mV/dec	30 nM–1 mM	V_g_: 0.7 V V_ds_: 0.05 V	~100 s (est.)	Buffer: PBS Working pH: 7.2	No interference was observed from AA and UA	[99]
rGO-FET	H_2_O_2_	MoS_2_	1 pM	0.46%/dec	1 pM–100 nM	V_g_: 0.1 V V_ds_: 0.01 V	~1 s	Buffer: PBS Working pH: 7.4	HeLa Cells	[25]
FET	H_2_O_2_	Graphene-Cyt-c	0.1 pM	14%/dec	0.1–100 pM	V_g_: 1.75 V V_ds_: 0.001 V	<1 s	Buffer: PBS Working pH: 7.4	No interference from UA, AA, dopamine, and glutamate	[24]
***Others***										
SiO_2_-MOSC	Glucose	HRP-GOD	5000	1.76 nA/cm^2^M	<2 M	V_g_: 5 V	1200	Dry sensor so no need for a buffer solution	-	[70]
Polysilicon wire-ISFET	H_2_O_2_ & Glucose	APTES-SiNPs-UV treatment	32 pM	12 AmM^−1^cm^−2^	10^−10^–10^−3^ M	V_ds_: 5 V	NR	Tested solution volume: 0.03 pL (Dry sensor)	Serum	[73]

The sensor exhibited a good response to glucose concentrations from 0.1 to10 mM with a detection limit of 0.1 mM. The response time was estimated to be ~200–500 s and the sensitivity of the sensors was ~1.5 μA/mM from 0.1 mM to 2 mM of glucose concentrations. A similar graphene based sensor was also reported [78]. The measuring range of the sensor was 3.3–10.9 mM with a detection limit of 3.3 mM. The response time of the sensor was ~60 s which was faster than the previously reported FET [77]. Graphene based FETs can work both in the p-doped (V_g_ < Dirac point) or n-doped (V_g_ > Dirac point) regime, depending on the applied gate potential.

Another study demonstrated the use of graphene flakes along with chitosan to improve the sensitivity of a TFT with a platinum gate electrode [98]. The sensitivity was improved from 41 mV/decade (no graphene) to 370 mV/decade with graphene along with a two-order of magnitude improvement in the detection limit from 1000 nM to 10 nM. The improvement in the sensitivity of the device was a result of high conductivity of the graphene and the larger surface to volume ratio of the composite film. The measuring range of the sensor was from 10 nM to 1 μM and the response time of the sensor was ~500 s. The same group has demonstrated an improvement of the electrocatalytic activity of the graphene film by depositing platinum nanoparticles on the film [99]. The detection limit was enhanced from 10 μM (without PtNPs) to 0.03 μM (with Pt NPs) of H_2_O_2_ concentration. The sensitivity of the sensor was 91.7 mV/decade when the H_2_O_2_ concentration lies between 3–300 μM. The response time of the sensor was ~200 s. The same group combined graphene sheets with cytochrome c to further extend the detection limit of the FET sensor to the femtomolar range and a rapid response time of <1 s [24] (Figure 7e,f). The ultralow sensitivity (100 fM) of the sensor can be attributed to the high charge carrier mobility, large surface area and high conductivity of single layer graphene sheet. In addition, cytochrome c imparts the required specificity to the sensors for such low concentration measurements. The measuring range of the sensor was 100 fM to 100 pM with a sensitivity of 16% current change per decade of concentration change.

Another ultrasensitive H_2_O_2_ FET sensor was fabricated using r-GO with MoS_2_, another 2D material [25]. MoS_2_ acts as a catalytic layer to catalyze the conversion of H_2_O_2_ and imparts the required sensitivity for selective H_2_O_2_ detection. The measuring range of the sensor was 1 pM to 100 nM with a sensitivity of 0.46% current change per decade change in H_2_O_2_ concentration which was far lesser than the graphene based sensor reported by Lee et al. [24]. The response time was around 100 s.

Carbon based nanomaterials offer promising properties for ultra-sensitive H_2_O_2_ FET sensors. The sensing surfaces exhibit several advantages such as excellent selectivity, simple immobilization processes for enzyme attachment, and low-cost materials. The surfaces fabricated using graphene have superior properties which can be attributed to effective and uniform enzyme functionalization due to the flat surface of graphene, a functionalization that could change the number of tube-to-tube contacts for CNTs, and reduced sensitivity of CNT films due to the presence of metallic CNTs [77].

## 6. Outlook

An ideal sensor should have high accuracy, selectivity, specificity, reproducibility, and environmental stability. To address selectivity and specificity, both enzymatic and non-enzymatic methods are used to develop H_2_O_2_ sensors. Enzymatic sensors use peroxidase enzymes to measure the H_2_O_2_ concentration and exhibit excellent selectivity along with good sensitivity due to high affinity for the analyte. However, these sensors require extra fabrication steps for efficient enzyme immobilization on the substrate without affecting the enzyme activity. Multiple immobilization processes are used to preserve the activity of the enzyme while immobilizing it on the electrode. However, in most immobilization processes a significant reduction in enzymatic activity is observed. Further, the activity of the enzymes is also affected by the environmental conditions which limit the use of enzymatic sensors under specific conditions and defined shelf life. Limited studies have attempted to improve the enzyme stability. One such study used silk fibroin as the immobilizing matrix to improve the enzyme stability [79]. More studies are required to improve the long-term stability of the enzyme under various environmental conditions.

Considerable effort has been made to develop non-enzymatic sensors and artificial enzymes. Non-enzymatic sensors utilize numerous compounds with peroxidase-like activity such as Prussian blue, nanomaterials, metal oxide particles like MnO_2_, SnO_2_, TiO_2_, ZnO etc., perovskite oxides, and others. Unlike natural enzymes, catalysts are stable in environmental conditions and less expensive. These catalysts can be more robust and tailored according to the required fabrication process and applications. Future developments in this field will focus on overcoming the limitations of existing sensors and extending their application to new areas. Some of these developments have been identified and discussed below.

### 6.1. Contact Resistance and Its Engineering

Contacts are the connection between the material like graphene, CNT, etc. and the electronics output. The contact resistance between the 2D materials and metal limits the scalability and the device performance due to the presence of a Schottky barrier. Unfortunately, less work has been done to reduce the contact resistance for these FETs. One of the techniques used to reduce the contact resistance is to change the contact metal to a lower work function metal. For instance, using scandium with MoS_2_ reduced the Schottky barrier height to 30 meV and the contact resistance of the FET to 0.65 kΩ/μm [105,106]. Another strategy used to reduce the contact resistance is by using a metal which can interact strongly with the 2D material layer. Mo metal contacts with MoS_2_ reduced the contact resistance. However, this method is not suitable for all metals or 2D material layers [106]. Further work needs to be done to improve the scalability and reproducibility of the contact electrodes especially when flexible sensors are manufactured.

### 6.2. Real Sample Testing

Most of the reported sensors have demonstrated good sensor selectivity and specificity for H_2_O_2_ measurements in defined sample matrices like buffers. However, these sensors could face challenges when tested in real sample matrices like blood, sweat, urine, cell media etc., due to the presence of other interfering analytes present and through non-specific adsorption. Therefore, selectivity becomes a more important parameter than other sensor parameters such as response time, sensitivity etc., due to this complex environment. Further, the sensor should exhibit the required LOD and sensitivity under the given environmental conditions. The desirable sensor properties also vary depending on the final applications. For instance, cell viability measurements require the detection of H_2_O_2_ at sub-micromolar concentrations while H_2_O_2_-based lactate sensors work within the millimolar range.

Chemiresistive H_2_O_2_ sensors work mostly within a millimolar concentration change with one exception which worked till nanomolar. These sensors also have a response time within a range of 3–400 s which makes them a good low-cost alternative for H_2_O_2_ measurements in multiple areas. 3 out of 7 studies reported here have performed interference testing [19,20,49]. One of the device was exposed to common interfering molecules present in blood such as ascorbic acid, uric acid, acetoaminophen [49]. Other device was tested against common sugars like sucrose and fructose [19]. Third device was exposed to ethanol, methanol and acetone to investigate the interference from these molecules. Therefore, there is a need to do exhaustive interference testing with common interferents present in the different samples like blood, urine, and environmental samples. Only one sensor was tested with a real sample like juice, and iced tea [19]. For commercial success, the sensors should be tested in real samples. Moreover, chemiresistive sensors suffer from nonspecific adsorption which can be prevented either by sample preparation or coating the sensor surface to increase the selectivity of the sensors.

Conductometric sensors have a more diverse measuring range where a few sensors have measuring ranges similar to the chemiresistive sensors (millimolar range) while others can measure H_2_O_2_ concentrations as low as 0.01 ng/mL. However, the sensors had longer response times (5–30 min). These sensors were used to measure H_2_O_2_ in relatively wider ranges for applications including food manufacturing and processing, beverages, and immunoassays. Four out of eight studies reported were tested in real samples like human serum [21,58], alcoholic beverages [55], and yogurt [57]. The sensor response is strongly affected by the ionic strength of the sample. Therefore, these sensors need sample dilutions or a preparation step for the measurement. Recent studies are working towards coatings which can reduce the effect of buffer strength on the sensor response.

FETs have the lowest reported LOD of 0.1 pM and a broad range of response times ranging from <1 s to 30 min for H_2_O_2_ measurements. This sensor group was tested with a wider range of samples including blood, serum, live cell cultures etc. These sensors also have broad measuring range where a few sensors worked in the millimolar range and others worked down to picomolar concentrations. Recent studies showed no significant effect of interfering species like ascorbic acid, uric acid, dopamine and glutamate. FET based sensors can be good candidates for real time H_2_O_2_ monitoring. Therefore, studies should focus more on testing the sensors in real samples and the long-term effect of the sample matrices on the sensor performance.

### 6.3. In Vivo Applications

In vivo H_2_O_2_ monitoring is useful for applications such as medical diagnostics, food processing industries etc. At present, none of the solid-state sensors are used for in vivo monitoring. Key factors crucial to consider while designing an in vivo sensor for reliable measurements are selectivity, and compatibility with the biological matrix. Selectivity is the most important parameter for sensors to generate accurate results. Unlike in vitro conditions, in vivo applications pose challenges due to the presence of coexisting compounds and dynamic environmental conditions. For example, in vivo detection of H_2_O_2_ in the human body can be affected by the presence of generated metabolites such as ascorbic acid, uric acid and others. To attain selectivity under such conditions, the reaction kinetics must be modified to ensure that the target analyte reaction is the favorable reaction. Selectivity can also be attained by using highly selective active molecules like enzymes or enzyme mimics for detection. The enzyme FET sensor is a good candidate for continuous H_2_O_2_ monitoring because FETs can be mass manufactured and miniaturized to accommodate multiple sensors on a same chip. However, FETs are currently affected by high drift rates and poor electrical insulation for the electrical contacts. The sensors can be modified using selective membranes to reduce the interference from coexisting compounds. A second factor that needs to be considered is compatibility of the sensor in the matrix. For instance, a sensor can give unpredictable results if its surface is fouled when placed in the sample matrix. The compatibility of the sensor is generally improved by reducing size which reduces the sensor exposure to the sample, but this can also impact the sensor sensitivity. To improve sensitivity, the sensing surface is often coated with antifouling materials that may be composed of nanomaterials such as metal nanoparticles. Sensor fouling can affect the sensor response both in terms of specificity and sensitivity. Researchers are exploring various physical and chemical methods to prevent fouling of the electrode surface. Physical methods such as membrane filtration, surface topographical engineering and chemical surface modifiers like polyethylene glycol are commonly used as anti-fouling strategies. A recent review has summarized various methods to prevent fouling in both in vitro and in vivo conditions [107]. Addressing the long term fouling issue will be critical in the effective demonstration of in-vivo application of these sensors.

## 7. Conclusions and Future Directions

The review has analyzed the performance of three types of solid state H_2_O_2_ sensors: chemiresistive, conductometric and FET. These sensors showed a good potential for low-cost, and easy to manufacture H_2_O_2_ measurement systems. Further, a wide range of new materials, including metal nanoparticles, carbon nanomaterials, and other materials, are also used to increase the sensitivity of these solid-state sensors and to reduce the potential applied for the measurements. Recent studies have also investigated the effect of common interferents on the sensor’s response. However, there is still a need to conduct more exhaustive studies to test these sensors in real samples and subsequently for in vivo monitoring of H_2_O_2_.

H_2_O_2_ is an important molecule in various industrial applications like textile, pharmaceuticals, food processing, cleaning and disinfection. Solid state sensors can work in a wide range of detection as summarized. However, these sensors still need improvement in sensitivity and selectivity for H_2_O_2_ in various sample matrix to become widely applicable in industrial applications mentioned above. Nanomaterials like graphene, CNT, and graphene oxide are used to improve the sensor performance in environmental conditions. More efforts will be needed to tackle the sensor fouling issues in different sample matrix to enable continuous monitoring required for industrial applications. Recent studies are working to reduce the limit of detection using FET based sensors.

In summary, solid state sensors could potentially be used for different applications due to simple fabrication process, wider range of detection and low-cost. Chemiresistive and conductometric sensors can be good alternatives to electrochemical sensors as they do not require reference electrodes which poses a challenge for miniaturization of amperometric and potentiometric electrochemical sensors.

## Figures and Tables

**Table 2:** Summary of H_2_O_2_ conductometric sensors with the crucial sensor properties including LOD, measuring range, voltage bias, response time, buffer and working pH. Where AOX: Alcohol oxidase, PVA: Polyvinyl alcohol, g-C_3_N_4_: graphitic carbon nitride, Au: Gold, AuNPs: Gold nanoparticles, AFP: alpha-fetoprotein, LOD: Lactate oxidase.

Substrate	Target Analyte	Ligand/ Enzyme	LOD (µM)	Measuring Range (mM)	Voltage Bias (mV) (Frequency)	Response Time (Minutes)	Buffer/ Working pH	Comments	Interference Tested	Ref
***Metal interdigitated electrodes***										
Ceramic-Au	H_2_O_2_	Pthalocyanine	NR	0.005–0.3	60	10	Working pH 6.0 Buffer: Phosphate (20 mM)	Storage stability for 90 days at 4 °C	No	[43]
Silicon-Au	H_2_O_2_/ Cyanide	PVA-Catalase	6	0–100	10 (100 kHz)	5	Working pH 7.2 Buffer: Phosphate (5 mM)	Inhibitory assay for cyanide detection	No	[56]
Au	Methanol	AOX-Catalase	0.5	<0.075	10 (100 kHz)	<10	Working pH 7.2 Buffer: Phosphate (5 mM)	Alcoholic beverages	Yes	[55]
	Ethanol		1	<0.070						
	Propanol		3	<0.065						
Ceramic-Au	Lactate	LOD-HRP	0.05	0–0.21	10 (100 kHz)	~20 (est.)	Working pH 6 Buffer: Phosphate (5 mM)	Diluted yogurt samples Storage stability for 40 days at 4 °C	Yes	[57]
***Metal nanoparticles***										
AuNPs	Hepatitis B (HB)	HRP/Anti-HBs	0.01 ng/mL	0.1–600 ng/mL	10 (100 kHz)	>30	Working pH 7.0 Buffer: Phosphate (10 mM)	Tested with serum samples Assay stable for 16 days when stored at 4 °C	Yes	[58]
Ceramic-Au & magnetic NPs	Glucose	GOD	3	0.04–3	10 (100 kHz)	<10	Working pH 7.3 Buffer: Phosphate (5 mM)	Stable for 12 days when stored at 4 °C	No	[23]
g-C_3_N_4_	AFP/H_2_O_2_	Pt NPs	0.01 ng/mL	0.01–100 ng/mL	10 (100 kHz)	5–6	Working pH 6.5 Buffer: PBS (10 mM)	Tested with human serum Inhibitory Immunoassay	Yes	[21]
***Others***										
Cellulose- SnO_2_	H_2_O_2_/ Glucose	GOD	500	0.5–12	0–3 V (dc)	NR	Working pH 7.2 Buffer: Phosphate	Storage stability > 10 days Flexible one-time use sensor	No	[59]

## Data Availability

Data is contained within the article or supplementary material.
